# Regional Elastic Properties of the Achilles Tendon Is Heterogeneously Influenced by Individual Muscle of the Gastrocnemius

**DOI:** 10.1155/2019/8452717

**Published:** 2019-11-03

**Authors:** Jiping Zhou, Jiafeng Yu, Chunlong Liu, Chunzhi Tang, Zhijie Zhang

**Affiliations:** ^1^Clinical Medical College of Acupuncture, Moxibustion, and Rehabilitation, Guangzhou University of Chinese Medicine, Guangzhou, China; ^2^Department of Rehabilitation Medicine, The First Affiliated Hospital of Zhengzhou University, Zhengzhou, China; ^3^Luoyang Orthopedic Hospital of Henan Province, Orthopedic Hospital of Henan Province, Luoyang, China

## Abstract

**Background:**

Anatomical studies and the mechanical property studies showed that there is a strong correlation between Achilles tendon (AT) elasticity and individual gastrocnemius muscle (the medial head of gastrocnemius (MG) and the lateral head of gastrocnemius (LG)) elasticity. Limited ankle dorsiflexion range of motion has been correlated with decreased flexibility of the MG/LG/AT complex. However, no studies have been conducted to examine the exact correlation between the Achilles tendon and the individual muscle of the gastrocnemius.

**Purposes:**

The purposes of the present study were (1) to evaluate intra- and interoperator reliabilities of elastic property measurements in the gastrocnemius muscle-Achilles tendon complex by using the shear wave elastography (SWE) and (2) to examine the correlation between the regional elastic properties of the AT and the individual muscle of the gastrocnemius.

**Methods:**

Twenty healthy subjects (mean age: 22.50 (3.02) years) were recruited in this study. The elastic properties of the AT and the individual muscle of the gastrocnemius were quantified using the SWE.

**Findings:**

The SWE has comparatively high reliability in quantifying the elastic properties of the muscle-tendon range from good to excellent. The intraoperator ICC of the gastrocnemius muscle-Achilles tendon complex was 0.77 to 0.95, while the interoperator ICC was 0.76 to 0.94. The minimal detectable change (MDC) of the muscle was 1.72 kPa, while the AT was 32.90 kPa. A significant correlation was found between the elastic modulus of AT and the elastic modulus of the MG (*r* = 0.668 and *p* = 0.001 at the relaxing position and *r* = 0.481 and *p* = 0.032 at the neutral position).

**Conclusions:**

The SWE has the potential to assess localized changes in muscle-tendon elastic properties, provide more intuitive relations between elastic properties of the muscle tendon and function, and evaluate the therapeutic effect of the muscle tendon. A significant correlation between the AT and the MG was found, and it may provide a new treatment idea (targeted to the tight muscle heads) for the clinical setting to treat subjects with AT disorders.

## 1. Introduction

The Achilles tendon (AT), which is the conjoined tendon of the gastrocnemius and soleus muscles, is the strongest and largest tendon in the body [1, 2]. Anatomical studies describe the Achilles tendon as the tendon that starts at the musculotendinous junction of the soleus and gastrocnemius, which becomes rounded and flattened in the junction with the gastrocnemius [3]. The twisted structure of the Achilles tendon is formed by individual subtendons which instead rotate as they descend but do not run in parallel to each other [4]. In addition, the posterior part (i.e., superficial part) of the Achilles tendon is formed by the fascicles of the tendons of the medial gastrocnemius (MG), while the anterior part (i.e., deep part) consists of the lateral gastrocnemius (LG) and soleus muscles [5]. Furthermore, the LG is shorter and smaller than the MG so that the lateral head and the medial head of the gastrocnemius vary in their force contribution to the Achilles tendon [3]. It has been reported that flexibility is a component of joint fitness, as it is thought to be associated with athletic performance and the incidence of injury [6, 7]. Note that in all the previous studies, flexibility was assessed by measuring the maximal range of passive joint motion [8, 9]. However, the passive range of joint motion depends on muscle flexibility or, alternatively, tendon extensibility. However, previous studies did not conduct studies of the individual muscle of the triceps calf muscle, and current research is still focused on mixed muscles or group muscles. Besides, Green et al. found that muscle heads of the soleus versus the gastrocnemius may differ when exercise induced passive muscle tension on the muscle [10]. In summary, there is a strong correlation between the Achilles tendon and the gastrocnemius. However, no studies have been conducted to examine the exact correlation between the Achilles tendon and the gastrocnemius.

The main function of the AT is the plantar flexion of the ankle, determining the human body's jumping and running [11]. Joint flexibility is influenced by musculotendinous structures around the joint. The tendinous tissues are known to possess elastic characteristics which are a critical determinant of the proper muscle force transmission and movement generation [12]. Muscle elasticity is the key determinant of the capacity of the muscle to contract [13]. Although there are different functions between the skeletal muscle and the tendon, modulation in the elastic properties of the muscle tendon can reflect the pathological change and recovery effect. In our previous study, we have explored the interplay between passive muscle tension of the vastus lateralis and rectus femoris muscles and patellar tendon stiffness. Greater passive tension in the vastus lateralis was associated with higher patellar tendon stiffness in male athletes. In addition, previous studies have investigated that stiffer AT and gastrocnemius have been found to be risk factors associated with Achilles tendinitis [14–17]. Reduced flexibility of the gastrocnemius muscle and the AT can lead to an increase in lower-extremity injuries. Increasing the stiffness of the muscle tendon could limit the range of joint motion and increase the risk of injury [18]. Moreover, Gajdosik et al. found that the flexibility of the ankle joint depends on the extensibility of musculotendinous structures, especially the gastrocnemius [19]. Similarly, the gastrocnemius-Achilles tendon unit has been suggested to be a major contributor to passive joint stiffness [20]. Both of them cannot separate the muscle-tendon unit into respective components. Thus, accurately quantifying the elastic properties of isolated regional areas of the MG, LG, and AT may provide a more comprehensive understanding about the biomechanics of the MG, LG, and AT.

Shear wave elastography (SWE) is a noninvasive technique to quantify the elastic properties of soft tissues. It can be used to estimate the elastic modulus of the regional area through the shear wave speed. The principle of SWE is (1) to create a shear wave through acoustic radiation force, (2) to use sonography to map the distortion induced by the wave in the measuring object, and (3) to trace the wave back to the mechanical properties of the measuring object by using the relationship *E* = 3*pc*_*s*_^2^ [21]. The SWE was used to quantify the elastic properties of the skeletal muscle and tendon. The stiffer the tissue, the greater the shear modulus (kPa) [22]. Our previous studies demonstrated that the SWE is a valid and reliable tool to estimate the elastic properties of the tendon. There is a significant correlation between the elastic modulus of the tendon captured from SWE and the tangent traction modulus computed from a material testing system [23]. In addition, Eby et al. revealed that the SWE is valid technique to estimate the elastic properties of the muscle. A positive significant correlation was found between the elastic modulus of the muscle from the SWE and the elastic modulus from a material testing system [24]. Furthermore, the SWE was also used to examine the elastic properties of the infraspinatus, erector spinae, and gastrocnemius muscles, rotator cuff, sternocleidomastoid, Achilles tendon, and patellar tendon [25–29]. Thus, the SWE can provide an opportunity to monitor the change in the elastic modulus of the gastrocnemius and the Achilles tendon at various angles of the ankle joint.

The objectives of this study were (1) to evaluate intra- and interoperator reliabilities of the elastic modulus measurement in the gastrocnemius muscle-Achilles tendon complex by using the SWE and (2) to examine the correlation between the regional elastic properties of the Achilles tendon and the individual muscle of the gastrocnemius during various angles of ankle joint flexion.

## 2. Methods

### 2.1. Ethics Statement

This study was approved by the Human Subjects Ethics committee of the Clinical Medical College of Acupuncture, Moxibustion, and Rehabilitation. All subjects were fully informed of the purpose and experimental procedures and signed the informed consent before the study.

### 2.2. Participants

Twenty healthy subjects (14 males and 6 females; age: 18–28 years) were recruited in this study. Inclusion criteria were that all participants were healthy and did not have a history of ongoing neuromuscular diseases or musculoskeletal injuries specific to the ankle or knee joints during the previous six months. Exclusion criteria included pain in the gastrocnemius and Achilles tendon, participants taking fluoroquinolone antibiotics, lower limb fracture, postoperative rupture of the Achilles tendon, or anomaly on ultrasound.

### 2.3. Equipment and Parameter Setting

An ultrasound shear wave elastography system (Aixplorer Supersonic Imagine, France) with a 40 mm linear array transducer (SL10-2, Supersonic Imagine, France) was used. The settings of the SWE system were set as follows: the instrument was set in the musculoskeletal mode. The frequency was 2~10 MHz. The SWE Opt was the penetration mode. The opacity was 85%. The elastic modulus range of the gastrocnemius was 0~200 kPa, while the elastic modulus range of the Achilles tendon was 0~800 kPa. The color scale used in the shear modulus (in kPa) showed the lowest values in blue to the highest values in red. The depth of the B-scan was 3.0 cm [30]. For the Achilles tendon, the size of the regions of interest (ROI) had to be set to 25∗12 mm and the Q-Box™ diameter was defined by the thickness of the tendon, which was the distance between the superior and inferior borders of the Achilles tendon [23]. For the MG and LG, the size of the ROI had to be set to 10∗10 mm and the diameter of the Q-Box™ is 5∗5 mm [31]. The transducer was positioned along the longitudinal axis of the AT, MG, and LG.

### 2.4. Experimental Design and Protocol

#### 2.4.1. Measurement Position

**Figure 1:**
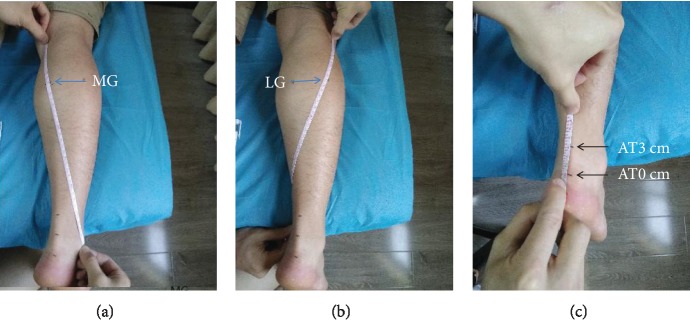
The location of the measurement site. (a) MG: at the midpoint of the oblique line connecting the inside of the popliteal transverse line and the highest point of the malleolus lateralis. (b) LG: at the midpoint of the oblique line connecting the outside of the popliteal transverse line and the highest point of the malleolus medialis. (c) AT0cm: 0 cm above the calcaneal tuberosity; AT3cm: 3 cm above the calcaneal tuberosity.

The dominant leg was identified by kicking a ball [32]. Before testing, subjects were asked to wear loose-fitting clothes. During testing, each subject was allowed to lie in the prone position with the foot relaxed and hung over the lower edge of the treatment bed, the hip and knee joints fully extended, and the upper limbs placed on both sides of the body [32]. The joint was fixed using a customized and movable ankle foot orthosis (AFO) at the neutral anatomical position (the strap of the AFO was tightened until a 90° ankle joint position was achieved, measured by a hand-held goniometer [33]) and resting ankle angle (participants took what they perceived to be a “relaxed” foot position [34, 35]). The measurement site of the Achilles tendon was defined as 0 cm (the most proximal outline of the calcaneus) and 3 cm above the calcaneal tuberosity [36], while the MG and LG were defined as the proximal 30% of the lower leg length [37]. The length of the MG is that measured from the popliteal fossa to the lateral malleolus, where cross-sectional areas of the gastrocnemius are almost maximum. As for the LG, the length is that measured from the popliteal fossa to the medial malleolus. The length was measured by a tape measure, and a black pen was used to mark the location of the measurement site (Figure 1). In addition, to reduce experimental errors, subjects were explicitly asked to keep the lower extremity as fully relaxed throughout the duration of testing and refrain from high intensity exercise for 48 hours before testing [32]. Room temperature was maintained at 25°C to reduce the effect of temperature on the elasticity of musculature [38].

#### 2.4.2. Ultrasound Shear Wave Elastography

**Figure 2:**
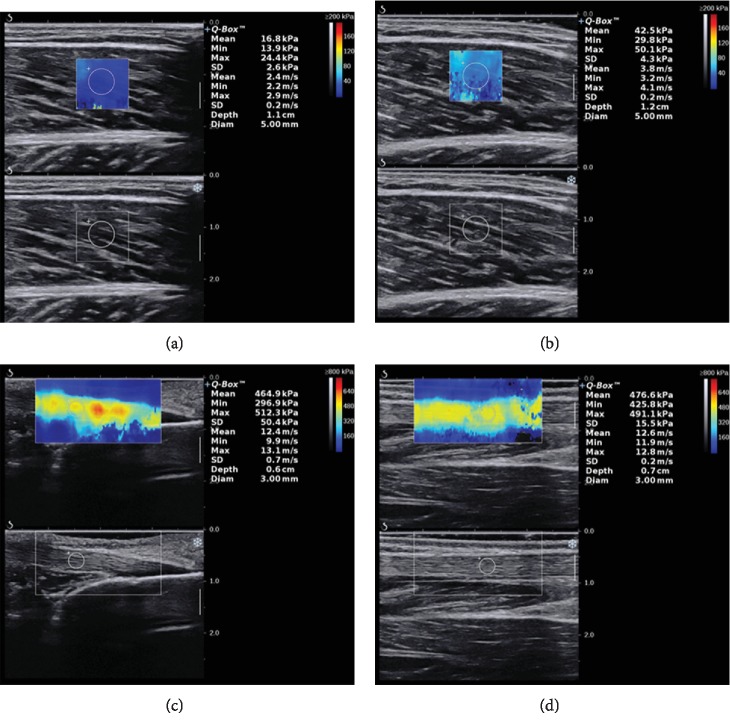
Changes of the elastic modulus of MG converted from the resting ankle angle to the neutral anatomical position (from (a) to (b)). The elastic modulus of AT0cm and AT3cm in the resting ankle angle (from (c) to (d)). The color-coded box presentation of muscle-tendon elasticity is shown in the upper images. The longitudinal grey scale sonograms of the muscle tendon are shown in the bottom images. The Q-Box™ is shown on the right.

Before measurements, subjects were asked to relax for 5 min to ensure that the triceps surae was relaxed. During measurements, enough ultrasound gel was applied between the skin and the transducer to avoid skin deformation. The midpoint of the transducer was placed perpendicularly on the skin's surface with a light pressure where we marked before, and then the mode of the SWE was activated to examine the shear wave modulus of the muscle or tendon [32]. During the acquisition of the mode of the SWE, the transducer was kept motionless for about 5-8 s [23]. Then, the gray scale image showed the appearance of the muscle or tendon under the longitudinal section. Image quality was closely monitored throughout the measurements. When the color in the ROI was uniform and several muscle fibers or the superior and inferior borders of the tendon were continuously visible, the images were frozen and then put on the Q-Box™ to obtain the shear wave modulus from the system and stored for off-line analysis (kPa) (Figure 2). Three images were captured at each measurement site of the tendon and muscle. The mean of the elastic modulus from all 3 images was used for further analyses.

Two operators (ZJP and ZJ) participated in the interoperator investigation. The operators took turns to examine each subject's Achilles tendon, MG, and LG over a 1-hour period and by operator ZJ with a 2-hour interval. In the second test, the same subjects attended at the same time 5 days later, which was repeated by operator ZJP for the intraoperator investigation. Subjects were explicitly asked to refrain from any additional exercise throughout the duration of testing but to maintain their normal daily walking activity [28]. The results were not communicated between operators ZJP and ZJ until all subjects had been examined.

#### 2.4.3. Statistical Analysis

Statistical analysis was performed using SPSS Version 19.0 (SPSS, Chicago, IL). All data were presented by the mean (standard deviation). The intraclass correlation coefficient (ICC) was calculated to determine the intra- and interoperator reliabilities. The coefficient of variance (CV), the standard error measurement (SEM), and the minimal detectable change (MDC) were calculated (all based on the following formulae: CV = (standard deviation/mean) × 100%, $\text{SEM}=\text{standard deviation}\times \sqrt{\left(1-\text{ICC}\right)}$, and $\text{MDC}=1.96\times \text{SEM}\times \sqrt{2}$). Pearson correlation analysis (*r*) was used to examine the correlation between the shear modulus of the Achilles tendon and the gastrocnemius muscle. The strength of reliability coefficients was classified as follows: excellent (more than 0.90), good (0.71–0.90), moderate (0.50–0.70), and poor (less than 0.50) [39]. The statistical significance was set at an alpha level of *p* < 0.05 (*α* = 0.05).

## 3. Results

### 3.1. Intra- and Interoperator Reliabilities

The intra- and interoperator reliabilities of the shear modulus exponent of the MG, LG, and Achilles tendon is shown in Tables 1 and 2, respectively. The results indicated that the intraoperator ICC for the MG, LG, AT0cm, AT3cm was 0.77 to 0.95 and the interoperator ICC was 0.76 to 0.94. The intraoperator reliability for the measurement site of the LG (relaxing position), MG (neutral position), LG (neutral position), and AT0cm (neutral position) is excellent. The interoperator reliability for the measurement site of the LG (relaxing position) and AT3cm (neutral position) is excellent. The rest of the ICC values reveal good intra- and interoperator reliabilities. The MDC (kPa) for the MG, LG, AT0cm, and AT3cm were 3.24 to 8.07, 1.72 to 6.44, 35.91 to 60.28, and 32.90 to 43.42, respectively.

### 3.2. The Relationship between the Shear Modulus of AT and MG and LG

**Figure 3:**
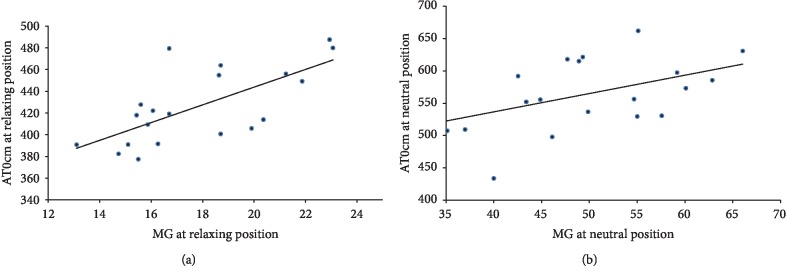
The plots show the correlations of AT0cm and MG at different angles. (a) Relaxing position: *r* = 0.668, *p* = 0.001; (b) neutral position: *r* = 0.481, *p* = 0.032.

The *r* values and *P* values of the AT and MG and LG for different ankle joint positions are shown in Table 3. A significant correlation was found between the AT0cm and MG (*r* = 0.668 and *p* = 0.001 at the relaxing position and *r* = 0.481 and *p* = 0.032 at the neutral position) (Figure 3). In addition, the correlation between the MG and AT3cm was weak (*r* = 0.146 at the resting position and *r* = 0.358 at the neutral position). Furthermore, no significant correlation was apparent in the shear wave modulus results between the LG and AT, in which the correlation coefficient is 0.078 to 0.199.

## 4. Discussion

The main findings of the present study were that the SWE had moderate to excellent intra- and interoperator reliabilities to quantify the elastic modulus of the gastrocnemius-Achilles tendon complex and a significant positive correlation was obtained between the elastic modulus of the AT0cm and MG, but not found for LG.

### 4.1. Intra- and Interoperator Reliabilities

The results of the present study demonstrated that the SWE has high intra- and interoperator reliabilities for quantifying the elastic modulus of the AT, MG, and LG. The relatively low SEM and MDC values revealed good precision of the measurements. The MDC is the smallest change in score that likely reflects true change (not measurement error alone). Comparing with those of previous studies, our study results revealed good to excellent intra- and interoperator reliabilities for quantifying the shear modulus of the Achilles tendon and gastrocnemius muscle. Chino et al. found that ultrasound elastography was a reliable and valid quantitative method for quantifying the stiffness of the gastrocnemius muscle, in which interoperator reliability was 0.77~0.89 [40]. Also, Fu et al. evaluated the elasticity of the normal Achilles tendon among 326 healthy subjects older than 18 years which were divided into different groups by age. The results of this study were that the intraoperator reliability was 0.93 [28]. In addition, Taş et al. made a study to examine the intra- and interoperator reliability levels for the stiffness of the rectus femoris muscle and patellar tendon. They found that the intraoperator reliability of the patellar tendon was 0.81~0.83 and the interoperator reliability was 0.71. For the stiffness of the rectus femoris muscle, the intraoperator reliability was 0.93~0.94 and the interoperator reliability was 0.95 [41]. Furthermore, Lima et al. found good intraoperator reliability between the AT and MG, bilaterally, during rest, in which the intraoperator reliability of MG was 0.98, while the AT was 0.82~0.93 [42].

In addition, a threshold of ≤12% for the CV is regarded as an acceptable level of biological measurement [43]. The results of the present study indicated that the CV of the MG and LG was 16.39% to 18.61%, which were a little different with the CV values of recent research. There is corroboration with the study by Lima et al. who observed values of 17.29% to 20.95% for the MG [42], while Chino and Takahashi observed values of 19.4% for the MG [44]. The possible reason is that we cannot ensure that the probe was completely parallel to the muscle fibers. However, the shear wave modulus values depend on the probe in relation to the fiber direction [45]. Brum et al. showed that the shear wave velocity dispersion was influenced by viscosity when the probe is perpendicular to the fibers, but it was not found parallel to the fibers [46]. Although the CV of the MG and LG in the present study was slightly higher than 12%, the ICC of the MG and LG values were 0.76 to 0.95, so the data of the muscle were all classified as acceptable. The CV of the Achilles tendon were 7.78% to 9.65%, which confirmed the high reliability of the shear elastic modulus values of the Achilles tendon, in agreement with a previous study (7.2% to 9.4%) [47].

Furthermore, the MDC was calculated to provide a value to reflect a real change that could be interpreted as a real difference exceeding the measurement error, which can serve as a reference for future study. Our results showed that the MDC of the muscle was 1.98 kPa (the same operator) and 1.72 kPa (different operator), while the Achilles tendon was 32.90 kPa (the same operator) and 39.86 kPa (different operator). Therefore, the shear wave modulus of the MG and LG should be greater than 1.72 kPa and the Achilles tendon should greater than 32.90 kPa to reflect changes with retested tests.

Recently, due to the high specificity and sensitivity of the SWE, it has been used in practical applications [48]. For example, Point et al. found that the SWE can monitor the increase of muscle stiffness after cryotherapy induction [49]. On the other hand, the SWE can be used to detect side-to-side differences in tendon geometry and mechanical properties in tendon structure in individuals with Achilles tendon rupture. This information may give clinical staff a clear understanding of the relationship between tendon structure and clinical manifestation [50]. Furthermore, it has been reported that the stiffness of congenital myopathies was changed. For example, the stiffness of cerebral palsy and Duchenne muscular dystrophy were increased, while decreased in GNE (UDP-N-acetylglucosamine 2- epimerase/N-acetylmannosamine kinase) [22, 51, 52]. Thus, the SWE can be used to quantify the shear modulus of the gastrocnemius muscle and Achilles tendon for assisting the clinical diagnosis (the muscle-tendon shear modulus of the normal side vs. the muscle-tendon shear modulus of the abnormal side) and treatment (pretreatment vs. posttreatment) [53]. Moreover, SWE has the characteristics of tracking the changes in the shear modulus of the gastrocnemius muscle and Achilles tendon. It may provide a basis to reflect a real change for examining the effectiveness of an intervention [50].

### 4.2. The Relationship between the Shear Modulus of AT and MG and LG

A significant positive correlation was obtained between the elastic modulus of the MG and AT, but not found for LG. This is the first study wherein a concurrent investigation of the gastrocnemius muscles and regions within the Achilles tendon has been undertaken. In the view of biomechanics, the transmission and generation of triceps surae muscle forces were affected by the compliance of the AT. Increasing AT compliance can alter the gastrocnemius muscle and soleus muscle fiber operating lengths and muscle excitations. And these effects in gastrocnemius muscles are more pronounced than in soleus muscles, which possibly arise from essential differences of muscle-tendon behavior. Gastrocnemius muscle fiber negative work decreased progressively with increasing AT compliance during the stretching phase while that of soleus muscles increased for 5% [54]. In addition, an imbalance of force generation between the MG and LG has also been speculated to contribute to the development and/or persistence of Achilles tendinopathy or the pain of the Achilles tendon [55]. On the one hand, both the MG and LG have the same function of plantarflexion but have different degrees of contribution, with the MG providing more than 70% of the muscle force [2]. On the other hand, the possible explanation is that the MG is longer and larger and extends more distally in the calf than the LG [3] and muscle volume of the MG was higher than the LG [56]. Furthermore, a study reported that the Achilles tendon has a unique structure that is the twisted descending structure, which enables it to handle the functional loads applied to the tendon [57]. Pękala et al. found that the torsion of the MG is significantly lower than the LG wherein the rotation angle of the LG is about 5 times that of the MG (the fibers originating from the LG rotate on average 135.98 ± 33.58° while the MG twist 28.17 ± 15.15°) [1]. In addition, the largest component of AT insertion into the calcaneal bone is the MG (the mean width of the footprint was 28.3 mm), and the smallest is the LG (the mean width of the footprint was 14.4 mm) [5]. In all the above views of biomechanics, the changes of the Achilles tendon are closely related to the MG, and the proportion of the MG contribution is larger than that of the LG contribution to the Achilles tendon.

The elastic properties of the Achilles tendon were closely related to the MG in various exercise programs. For example, during an acute bout of eccentric heel drop exercise, the gastrocnemius muscles were shown to bear larger mechanical loads than the Achilles tendon [58]. Stenroth et al. found that the longer distance walked in a 6-minute walk test was significantly associated with the MG [59]. Besides, Hirata et al. reported that the passive muscle stiffness differs among the triceps surae. They found a higher elastic modulus of the MG than the LG. After static stretching, a significant reduction in the elastic modulus of the MG was observed, but not found for the LG [60]. Masood et al. reported that there was a significant difference in activity of the MG and LG. The electromyography (EMG) was used to assess the activity of the MG and LG. They found an increase in the activity of the MG (34%) and LG (21%) during sustained submaximal isometric exercise [61].

Most studies have demonstrated there is a relationship between the gastrocnemius and Achilles tendon or foot function. Park et al. used the extracorporeal shock wave therapy on the medial head of the gastrocnemius muscle to relax the ankle in children with spastic cerebral palsy. They found that the passive range of motion of the ankle joint was significantly increased after ESWT (extracorporeal shock wave therapy) [62]. In addition, eccentric training is effective to reduce pain and improve function among subjects with Achilles tendinopathy. Crill et al. has reported that eccentric training could reduce pain and improve function among subjects with Achilles tendinopathy. They also found the fascicle length of the MG increased 12%, but not found for LG. The findings of the present study indicated that there is a greater response to eccentric training for the MG than the LG [63]. Based on previous studies, there is a strong correlation between the Achilles tendon and gastrocnemius. However, no studies have been conducted to examine the exact correlation between the Achilles tendon and gastrocnemius. Our study is the first to reveal a positive correlation between the elastic modulus of the Achilles tendon and the elastic modulus of the MG. Therefore, a reduction of tension of the MG was considered to be one effective method to prevent and treat the injury of Achilles tendon disorders.

### 4.3. Limitations

The present study has some limitations. Firstly, EMG was not used during the tests to monitor the muscle activity to ensure no contraction of the muscle. However, all participants were verbally instructed to stay relaxed, and no signs of muscle contraction were visible on the B-mode image. Based on this, we are confident that participants remained in a passive state while their legs were fully supported. Secondly, due to the limitation of the SWE, the soleus muscle was involved in this study. The SWE could not measure the deep muscle. Thirdly, the ankle of participants was placed in a “relaxed” position; muscle tension may be affected by the mass moment of inertia of the foot. Further studies will be conducted to investigate the modulation of elastic properties of the mass moment of inertia of the foot. Finally, only healthy subjects and only 2 angles of the ankle were recruited in this study. Further studies will be conducted to investigate the modulation of elastic properties of the Achilles tendon and gastrocnemius among individuals with Achilles tendon disorders.

## 5. Conclusion

The SWE can be used to quantify regional elastic properties of the Achilles tendon and gastrocnemius. We also found a significant correlation between the elastic modulus of the Achilles tendon and the elastic modulus of the MG, but not for the LG. These findings suggested that an increase in tension of the MG muscle may increase the tension of the Achilles tendon.

### 5.1. Perspectives

Different ankle angle positions notably affect the tension of the gastrocnemius muscle and Achilles tendon. The present study suggests a significant correlation between the elastic modulus of the Achilles tendon and the elastic modulus of the MG, but not for the LG. This finding may provide a new treatment idea for the clinical setting to treat subjects with Achilles tendon disorders. A reduction of tension of the MG may be considered to be one effective method to prevent and treat the injury of the Achilles tendon. This study was limited to participants who were healthy and did not have a history of ongoing neuromuscular diseases or musculoskeletal injuries specific to the ankle or knee joints. The same kind of experiment could be valuable to analyze the correlation between the elastic modulus of the Achilles tendon and the elastic modulus of the MG in Achilles tendon disorders. More research is needed to explore whether current management programs for Achilles tendon disorders need to be tailored to the MG.

## Figures and Tables

**Table 1:** Intraoperator reliability of mean elastic modulus values of MG, LG, and AT.

Measurement position	Test 1 (kPa)	Test 2 (kPa)	MDC (kPa)	95% CI	CV (%)	SEM (kPa)	ICC
MG (R)	17.82 ± 2.92	17.85 ± 3.47	3.24	0.61-0.94	16.39	1.17	0.84
LG (R)	14.19 ± 3.28	14.60 ± 2.53	1.98	0.79-0.97	17.33	0.72	0.92
AT0cm (R)	426.04 ± 34.62	422.83 ± 32.91	43.75	0.42-0.91	7.78	15.78	0.77
AT3cm (R)	415.19 ± 38.44	402.59 ± 35.75	42.04	0.54-0.93	8.88	15.17	0.82
MG (N)	49.49 ± 9.21	49.98 ± 9.50	7.66	0.78-0.96	18.61	2.76	0.91
LG (N)	39.14 ± 7.01	38.95 ± 6.71	4.16	0.88-0.98	17.91	1.50	0.95
AT0cm (N)	563.52 ± 54.37	568.53 ± 51.84	43.11	0.76-0.96	9.12	15.55	0.91
AT3cm (N)	529.07 ± 44.86	525.27 ± 41.51	39.86	0.71-0.95	7.90	14.38	0.88

R: relaxing position; N: neutral position; LG: lateral gastrocnemius; MG: medial gastrocnemius; AT: Achilles tendon; MDC: minimal detectable change; 95% CI: 95% confidence interval; CV: coefficient of variation; SEM: standard error in measurement; ICC: intraclass correlation coefficient.

**Table 2:** Interoperator reliability of mean elastic modulus values of MG, LG, and AT.

Measurement position	Operator ZJP (kPa)	Operator ZJ (kPa)	MDC (kPa)	95% CI	CV (%)	SEM (kPa)	ICC
MG (R)	17.82 ± 2.92	18.21 ± 3.96	3.97	0.39-0.90	16.39	1.43	0.76
LG (R)	14.19 ± 3.28	14.19 ± 2.54	1.72	0.84-0.97	17.90	0.62	0.94
AT0cm (R)	426.04 ± 34.62	428.83 ± 39.48	35.91	0.70-0.91	8.13	12.95	0.86
AT3cm (R)	415.19 ± 38.44	407.43 ± 32.66	43.42	0.43-0.91	8.02	15.66	0.77
MG (N)	49.49 ± 9.21	51.68 ± 9.42	8.07	0.76-0.96	18.61	2.91	0.90
LG (N)	39.14 ± 7.01	41.36 ± 7.87	6.44	0.73-0.96	17.91	2.32	0.89
AT0cm (N)	563.52 ± 54.37	565.39 ± 60.93	60.28	0.61-0.94	9.65	21.75	0.84
AT3cm (N)	529.07 ± 44.86	535.78 ± 42.13	32.90	0.84-0.97	7.86	11.87	0.93

**Table 3:** Relationships of the elastic modulus between the AT and MG and LG.

	AT0cm (R)	AT3cm (R)
MGR	0.668/0.001^∗∗^	0.146/0.540
LGR	0.09/0.970	0.078/0.745

	AT0cm (N)	AT3cm (N)
MGN	0.481/0.032^∗^	0.358/0.121
LGN	0.199/0.399	0.143/0.547

Data are *r* value/*P* value. R: relaxing position; N: neutral position. ^∗∗^*p* < 0.01 and ^∗^*p* < 0.05.

## Data Availability

The data used to support the findings of this study are available from the corresponding author upon request.

## Abbreviations

SWE: Shear wave elastography
AT: Achilles tendon
MG: The medial head of the gastrocnemius muscle
LG: The lateral head of the gastrocnemius muscle
AT0cm: The position of the Achilles tendon at 0 cm above the calcaneal tuberosity
AT3cm: The position of the Achilles tendon at 3 cm above the calcaneal tuberosity.
